# Accelerating quality midwifery education: identifying educators’ professional development needs

**DOI:** 10.1186/s12909-026-09322-4

**Published:** 2026-04-29

**Authors:** Frida Berg, Kerstin Erlandsson, Noor Islam Pappu, Joy Kemp, Pronita Raha, Helena Wigert, Rabeya Basri, Malin Bogren

**Affiliations:** 1 Institute of Health and Care Sciences, Sahlgrenska Academy, University of Gothenburg, Arvid Wallgrens backe, House 1, Gothenburg, 413 46 Sweden; 2 School of Health and Welfare, Dalarna University, Falun, Sweden; 3 United Nations Population Fund, Dhaka , Bangladesh; 4 Division of Neonatology, Sahlgrenska University Hospital, Gothenburg, Sweden; 5 Directorate General Nursing and Midwifery Government of the People’s Republic of Bangladesh, Dhaka, Bangladesh

**Keywords:** Midwifery education, Midwifery faculty, Cross-sectional survey, South-East-Asia, Quality assurance

## Abstract

**Background:**

The quality of midwifery education and the quality of care are influenced by the capabilities of those who educate future midwives. In Bangladesh, where midwifery is a new profession, it is crucial that midwifery educators are supported, prepared and equipped to teach in both academic and clinical settings. This study aimed to identify the professional development needs of midwifery educators in Bangladesh.

**Methods:**

A cross-sectional online survey consisting of closed and open-ended questions, and statements answered using a four- and five-point response scale, was conducted. A total of 118 individuals responded to the survey, of whom 113 met the inclusion criteria and were included in the final analysis. Data were analysed using descriptive statistics including frequencies and percentages and inferential statistics (Spearman’s rank-order correlation and ANOVA). Internal consistency was evaluated using Cronbach’s alpha coefficient. Responses to the open-ended questions were analysed using systematic text condensation.

**Results:**

Among 113 respondents, working as an educator the majority (78%) held a master’s degree. The most reported development needs were in midwifery theory (41.6%), face-to-face teaching (41.6%) and clinical simulation (39.8%). Research capacity was also a key area, with 38.1% reporting a need for support in scientific manuscript writing. Leadership and management development were prioritised by 37.2%. Educators preferred face-to-face programme delivery (53.1%) and short, intensive formats (74%). Open-ended responses underscored a need for structured pedagogical training, as well as research and leadership mentorship.

**Conclusions:**

Continuous professional development for educators in Bangladesh is needed, particularly in education, research, and leadership. To address these needs, the study highlights the importance of incorporating its identified needs into a national educator development programme. We propose co-creating such a programme, guided by the ICM Global Standards for Midwife Faculty Development, to ensure a structured, relevant, and sustainable response.

**Supplementary Information:**

The online version contains supplementary material available at 10.1186/s12909-026-09322-4.

## Background

Midwifery educators’ capabilities play a foundational role in preparing competent, autonomous professionals who can deliver evidence-based, respectful care to women, newborns, and families [1–3]. Evidence links a strong midwifery profession to progress toward Universal Health Coverage and improved maternal and newborn health outcomes, particularly in low- and middle-income countries [4]. Ensuring that educators have up-to-date clinical expertise, strong pedagogical skills, leadership capacity, and professional support is crucial for maintaining and improving the quality of education and care [3, 5]. Educator development, encompassing systematic enhancement of pedagogical, clinical, research and leadership competence, is therefore recognized as a critical strategy to strengthen midwifery education and advance maternal and newborn health outcomes globally [4, 6].

In many low- and middle-income countries, midwifery educator professional development remains under-addressed despite repeated global calls for investments [7, 8]. Persistent challenges include unclear career pathways from clinical to academic roles [9] and weak systems, such as resource constraints, challenges in curriculum implementation, and insufficient pedagogical and mentorship, to support continuing growth and clinical practice [9–11]. An integrative review of midwifery education in South-East Asia found that many educators lack formal qualifications, receive inadequate pedagogical preparation and are appointed without relevant academic credentials, patterns reported in Bangladesh, India, and Nepal [2]. These gaps constrain educator capacity in classrooms and clinics, with predictable downstream effects on graduate competence.

Systemic constraints further erode educators’ ability to sustain competence [2, 12, 13]. Recent evidence from South-East Asia highlights limited formal preparation for educator roles, competing responsibilities that dilute time for teaching and few opportunities to maintain clinical skills [12, 14]. National mechanisms for continuing professional development are often insufficient or under prioritised, leaving development to individual effort rather than institutional policy [10, 15]. Since the government introduced the midwifery programme in Bangladesh in 2010, there have been several efforts to strengthen the midwifery educators [16–20]. Despite this, challenges remain, with 25% of public midwifery institutions reporting difficulties in providing sufficient clinical practice opportunities, limiting students’ ability to develop their skills and, in turn, affecting the reputation of the teaching educators [21]. The status of midwifery education in Bangladesh illustrates both momentum and risk. Over the past decade, the country has expanded midwifery education to nursing and midwifery educational institutions and integrated midwives into the health workforce [16] supported by the introduction of internal quality assurance processes to raise educational standards [13, 22]. However, without investment in educator preparation, mentorship and leadership development, these gains may not translate into durable improvements in graduate readiness or service quality [13]. Regional analyses indicate that while pre-service education is expanding, in-service educator development, including curriculum design, assessment literacy and academic leadership, remains under-resourced and under-researched [9].

Converging evidence from scoping reviews and qualitative studies reinforces the need for context-specific, career spanning educators’ development models that provide mentorship, clarify roles and build pedagogical and clinical capacity [9, 14, 23]. Taken together, these gaps justify a targeted assessment to inform policy and programme design. Accordingly, this study aimed to identify the professional development needs of midwifery educators in Bangladesh, generating evidence to guide tailored initiatives aligned with international standards and quality efforts.

## Methods

### Study design

A cross-sectional online survey consisting of closed and open-ended questions, and statements answered using a five-point response scale [24] was used. Approval in the form of a government order to conduct the survey with government employees was granted by the Directorate General of Nursing and Midwifery (DGNM) in 2024.

### Setting

Midwifery education in Bangladesh is currently offered through 171 educational institutions nationwide, comprising 62 public and 109 private institutes. These programmes follow a standardised three-year diploma curriculum, with a 60:40 ratio of clinical practice and theory. Annual student intake per institution ranges from 25 to 50, enrolling candidates from both urban and rural areas.

To enhance educational quality, the Government of Bangladesh has selected 31 institutions, both public and private, for implementation of an internal quality assurance process [22]. These efforts are supported by the United Nations Population Fund (UNFPA) Bangladesh, the Swedish International Development Cooperation Agency (Sida), and the University of Gothenburg. Their common aim is to strengthen midwifery education and the provision of services within each institution’s district.

### Study population

The educators were recruited from educational institutions where midwifery is offered as a distinct programme and where educators are responsible for teaching core midwifery subjects. Eligible respondents included those holding the title of *Nursing Instructor* or those employed in functional teaching positions formally designated for midwifery education. Staff midwives based in teaching hospitals and clinical supervisors were excluded. We employed a purposive sampling approach with 31 midwifery education institutions, 18 public and 13 private, invited across the country. By 2025, these 31 institutions employed a total of 349 staff, including an estimated 100 midwifery educators, although this figure may vary due to high staff turnover [25].

### Data collection

The recruitment of respondents was carried out in collaboration with DGNM and UNFPA. Prior to the recruitment, the research team conducted a 1-hour online webinar explaining the study and the data collection process, for principals and educators at respective institutions. Thereafter, e-mail invitations were distributed to eligible midwifery educators via DGNM, with follow-up by a national project coordinator. The e-mails included information about the study’s rationale, aim, methods, ethics and expected contributions of the survey results and a request to forward the invitation and survey link to other midwifery educator colleagues within their respective institution.

In May 2025, the data were collected through an online survey using a validated questionnaire previously developed to assess the professional development needs of midwifery educators [26, 27] and previously used in Africa [28]. For this study, the survey was adapted by the research team to reflect the Bangladesh context. The survey consisted of 33 multiple-choice items and included closed and open-ended questions, and statements answered using both four and five-point response scale (see Annex 1). Respondents were informed that their consent was implied through completion of the survey. A total of 118 respondent completed the survey of whom 113 met the inclusion criteria and were included in the final analysis.

### Data analysis

The data were entered into IBM’s SPSS™ Statistical version 29 and analysed using descriptive statistics [24], including frequencies and percentages. For the Likert-scale related to perceived development needs, responses indicating a highest level of need, a score of 5 on a 5-point Likert scale, were aggregated and reported as percentages across key thematic areas: education, research and leadership. Inferential statistics were used to analyse differences and relationships between variables. Internal consistency was assessed using Cronbach’s alpha, with values ≥ 0.70 considered acceptable. A composite development needs index was constructed by calculating the mean score across items 10–21 assessing professional needs (Annex 1). Spearman’s rank correlation was used to examine associations between the development needs index and continuous variables, including years of experience as a midwifery educator. Differences in the development needs index across categorical variables (e.g., profession and educational degree) were assessed using one-way analysis of variance (ANOVA). A *p*-value < 0.05 was considered statistically significant corresponding to a 95% confidence level. For the Likert-scale related indicate their most preferred delivery mode of a faculty development programme or module, a score of 4 on a 4-point Likert scale were analyse using percentage.

The 339 responses to the open-ended questions were analysed using systematic text condensation [29]. The research team read all responses to gain a general impression of the responses. Themes related to education, research and leadership were identified. The texts were sorted under each theme, condensed and described with illustrative quotes.

## Results

Among the 113 respondents, experience as an educator ranged from 1 month to 38 years (mean = 5.2 years). The majority were female, 93.8% (*n* = 106), and 6.2% (*n* = 7) were male. They had a mean age of 41.9 years, ranging from 24 to 72 years. A total of 46.9% (*n* = 53) reported working clinically alongside teaching, while 53.1% (*n* = 60) were exclusively engaged in theoretical teaching. Regarding educational qualifications, 69% (*n* = 78) held a master’s degree and 24.8% (*n* = 28) had a bachelor’s degree as their highest qualification. Furthermore, 58.4% (*n* = 66) had completed a formal educator development programme within the past five years, either through blended, online, or face-to-face formats. For more details see Table 1.

**Table 1:** Characteristics of the respondents

Respondent characteristics	*n* (%)
Gender	Female: 106 *(93.8)*
	Male: 7 *(6.2)*
Age	Minimum: 24
	Maximum: 72
	Mean 41.9 (SD = 12.1)
Profession	Certified Midwife: 24 *(21.2)*
	Midwife/Nurse-Midwife: 37 *(32.7)*
	Nurse: 41 *(36.3)*
	Other: 8* *(7.1)*
	Not accurate: 3 *(2.7)*
Highest education degree	Diploma: 3 *(2.7)*
	Bachelor: 28 *(24.8)*
	Master: 78 *(69.0)*
	PhD: 4 *(3.5)*
Year working as a midwifery educator	Minimum 1 month
	Maximum 38 year
	Mean 5.2 (SD = 6.1)
	Not accurate: 6
Multi-level-teaching	Diploma: 66 *(58.4)*
	Bachelor: 7 *(6.2)*
	Master: 9 *(8.0)*
	Diploma/Bachelor: 24 *(21.2)*
	Diploma/Bachelor/Master: 4 *(3.5)*
	Diploma/Bachelor/Midwifery: 2 *(1.8)*
	Not accurate: 1 *(0.9)*

* Principle, Master’s in Public Health, Master of Science in Nursing. Nursing instructor, Midwifery faculty

**Fig. 1:**
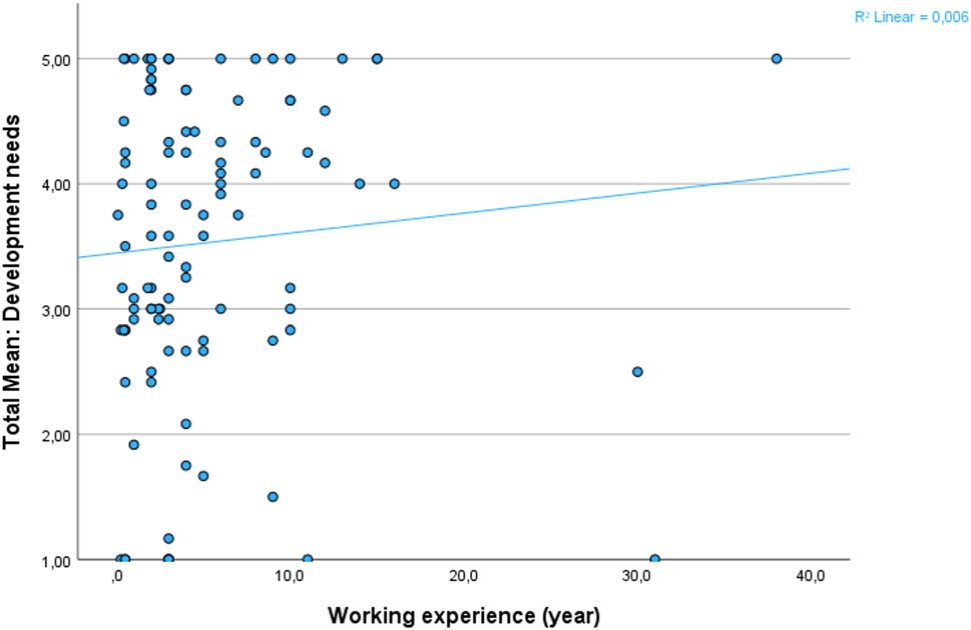
Scatter plot illustrating educators’ development needs (mean) in relation to years of work experience (index)

Overall, respondents more often chose higher Likert-scale ratings (4–5; 57.2%) than lower ratings (1–3; 42.8%) when assessing educators’ development needs (Table 2). The 12-item professional development needs scale demonstrated high internal consistency (Cronbach’s alpha = 0.963). Total scores ranged from 12 to 60, with a mean of 42.7 (SD = 14.36). Spearman’s rank-order correlation analysis showed a weak positive trend between years of experience as a midwifery educators and professional development needs index, although the association was not statistically significant (*ρ* = 0.155, *p* = 0.111) (Fig. 1). No statistically significant differences were observed by profession (F(4.105) = 0.17, *p* = 0.954), or educational degree (F(3.109) = 1.64, *p* = 0.184).

**Table 2:** Overview of reported professional development needs

Likert scale (1 = Very Low and 5 = Very High)	1	2	3	4	5
Face-to-face teaching	18.6%	11.5%	9.7%	18.6%	41.6%
Blend/Online teaching	13.3%	14.2%	20.3%	28.3%	23.9%
Clinical teaching using simulation	11.5%	8.9%	21.2%	18.6%	39.8%
Clinical teaching in the clinical environment	12.4%	9.7%	19.5%	21.2%	37.2%
Midwifery theory	11.5%	9.7%	20.4%	16.8%	41.6%
Midwifery clinical practice	11.5%	14.1%	18.6%	19.5%	36.3%
Curriculum design/Programme and module development	14.2%	6.2%	20.%	22.1%	37.2%
Conduct research	13.3%	12.4%	19.5%	22.1%	32.7%
Writing manuscripts for publication	17.7%	6.2%	16.8%	21.2%	38.1%
Management and leadership	15.9%	10.6%	18.6%	17.7%	37.2%
Educational quality improvement activities	11.5%	12.4%	17.7%	21.2%	37.2%

### Education

The results based on responses with Likert scale 5, reveal that educators expressed the greatest professional development needs within areas of education. The most frequently identified areas were midwifery theory, with 41.6% (*n* = 47) of respondents indicating a strong need for further competence, and face-to-face teaching 41.6% (*n* = 47). This was closely followed by clinical teaching using simulation 39.8% (*n* = 45). An equal proportion of educators 37.2% (*n* = 42) reported a strong need to strengthen their skills in both curriculum design and module development, as well as in clinical teaching within the clinical environment. Similarly, 36.3% (*n* = 41) indicated a high need for development in midwifery clinical practice and in mentoring and coaching capacities. The lowest reported area of need was blended or online teaching, noted by 23.9% (*n* = 27) of respondents. For more information see Table 2.

The open-ended questions identified a need for strengthened educational capacity, both at individual and institutional levels. There was a demand for development initiatives for educators, particularly in pedagogical content knowledge and practical clinical instruction. Educators reported for improved pedagogical training and enhanced infrastructure to support effective teaching. Comments highlighted limited teaching materials and opportunities for skill development. Two educators commented: *Teachers’ training is highly recommended for better education delivery.* (Respondent 59) //*As a midwifery faculty member*,* I believe ongoing training is essential to enhance our ability to educate effectively.* (Respondent 108). Many educators expressed a desire for structured refresher courses and standardized teaching aids, suggesting that quality improvement in midwifery education cannot be decoupled from investments in development for educators and educational infrastructure.

### Research

A need for research capacity development was evident, with 38.1% (*n* = 43) of respondents indicating a strong need to improve their ability to write scientific manuscripts for publication. Furthermore, 32.7% (*n* = 37) expressed a need to enhance their competencies in conducting research (Table 2).

The open-ended questions reflected the educators’ felt needs in scholarly production and methodological proficiency, including research design, analysis and academic writing. Comments by the educators revealed a demand for research utilisation for evidence-based midwifery practice as a core educator competency. Several comments alluded to the lack of engagement with research and the need for capacity building in academic scholarship. Respondents expressed interest in integrating evidence-based approaches into their work but reported few opportunities to participate in research training or collaborative projects. As commented by one educator: *Empower the faculty teachers and midwives in their respective roles. Develop the organogram and career path for midwives and faculty…Improve the research facility.* (Respondent 68)

### Leadership

Leadership and institutional development skills were also frequently endorsed as areas of high need. Specifically, 37.2% (*n* = 42) of respondents strongly agreed on the need to improve their management and leadership capacity. Similarly, 37.2% (*n* = 42) identified educational quality improvement activities as a priority area requiring enhancement. See Table 2 for more details.

The open-ended responses revealed a dual-level need for leadership capacity within midwifery education. At the individual level, educators highlighted the importance of targeted leadership development through formal training and mentorship; at the institutional level, they described the need for structured career pathways, supportive policies and strengthened cross-sectoral collaboration. Educators emphasised that institutional investment in professional development and quality assurance mechanisms is essential to support leadership growth across diverse educational contexts. There was a call for formal leadership training and the empowerment of educators to take on strategic roles within their institutions. As one respondent articulated:

*I think midwifery faculty should be proficient in leadership*,* theory*,* demonstration*,* evidence-based clinical practice and decision-making to work as midwifery faculty.* (Respondent 37)

Strengthening the educators´ leadership skills was viewed as critical to advancing sustainable and locally led reform in midwifery education.

### Educators´ development: preferred modes of programme delivery

Respondents were asked to indicate their preferred mode of programme delivery from the options: *Fully online*,* Fully face-to-face*,* Mostly online blended*, and *Mostly face-to-face blended*. The majority, 53.1% (*n* = 60) of educators, expressed a preference for the Fully face-to-face approach. This was followed by 41.6% (*n* = 47), who favoured the Mostly face-to-face blended format. Fully online delivery was preferred by 31% (*n* = 35), while 23.9% (*n* = 27) selected Mostly online blended, shown in Table 3. One respondent elaborated on this preference and commented: *Because eye-contact helps build trust and engagement during faculty development.* (Respondent 4)

**Table 3:** Preferred delivery modes among 113 midwifery educators

What would be your preferred delivery mode of a faculty development programme or module?				
Likert scale (1 = most preferred and 4 = least preferred)	1 *n* (%)	2 *n* (%)	3 *n* (%)	4 *n* (%)
Online only	35 (31.0%)	10 (8.8%)	15 (13.3%)	53 (46.9%)
Blend/Mostly online with limited face-to-face	27 (23.9%)	32 (28.3%)	32 (28.3%)	22 (19.5%)
Blend mostly/Face-to-face with limited online	47 (41.6%)	26 (23.0%)	29 (25.7%)	11 (9.7%)
Face-to-face only	60 (53.1%)	18 (15.9%)	10 (8.9%)	25 (22.1%)

What would be your preferred format/schedule for a XX-day or equivalent programme of faculty development?				
Likert scale (1 = most preferred and 4 = least preferred)	1 *n* (%)	2 *n* (%)	3 *n* (%)	4 *n* (%)
Single point-in-time 10 days intensive programme (e.g. over a couple of weeks)	74 (65.5%)	17 (15.0%)	9 (8.0%)	13 (11.5%)
Only one day each week for a number of weeks	36 (31.9%)	43 (38.1%)	13 (11.5%)	21 (18.6%)
Half day every two weeks over a period of 6 months	25 (22.1%)	17 (15.0%)	50 (44.3%)	21 (18.6%)
One day each month for e.g. 10 months	33 (29.2%)	11 (9.7%)	14 (12.4%)	55 (48.7%)

Regarding the preferred format and scheduling of educator development programmes 65.5% (*n* = 74) of respondents chose a single-point-in-time 10-day intensive programmed conducted over two weeks. Meanwhile, 31.9% (*n* = 36) of educators preferred a format of one day each week over several weeks. Other preferences included a half-day every two weeks over six months, favoured by 22.1% (*n* = 25) of educators and one day each month for ten months, chosen by 29.2% (*n* = 33) of educators (Table 3). As one respondent commented:

*As a midwifery faculty member with ongoing teaching and clinical duties*,* attending full-day sessions once a week is manageable without disrupting other responsibilities. The face-to-face sessions allow for practical*,* hands-on learning and interaction with peers and facilitators*,* while the online components provide flexibility to review materials*,* complete assignments and reflect at one’s own pace.* (Respondent 26)

## Discussion

This national survey identified three priority domains for strengthening quality midwifery education in Bangladesh through educator professional development: (i) pedagogical and curricula design capabilities, (ii) research and (iii) leadership competence. Despite most respondents having completed at least one educator development activity in the past five years, needs persist. These results underscore the need for continuous professional development. The newly released ICM Global Standards for Midwife Faculty Development [8] provide a guide for professional development. The guide consists of seven categories: (1) *Leadership*, (2) *Partnership and Collaboration*, (3) *Curriculum*, (4) *Learning and Teaching*, (5) *Resources* (6) *Maintaining midwifery practice competence* and (7) *Research* [8]. In this discussion section, we discuss the three-priority domain against these standards, while also situating our results within broader international literature on medical education and faculty development.

In core aspects of pedagogical and curriculum design capabilities, respondents in this study reported the strongest needs for more training in midwifery theory, clinical teaching and curriculum design. Strengthening these areas would enhance educators´ ability to apply sound pedagogical principles, integrate theory with practice, and design curricula that effectively support student learning and professional competence. These results are also confirmed by other studies [6, 30, 31] and directly address four of ICM´s global faculty standards: Category 3 *Curriculum*, Category 4 *Learning and teaching* and Category 6 *Maintaining midwifery practice competence* [8]. Despite investments in pedagogical training and educator development, many midwifery educators in Bangladesh still feel insufficiently prepared to teach according to international standards [17, 32]. As also noted in the State of the Asia´s Midwifery 2024 report [5], investment in educator development, especially in applied, context-relevant pedagogy, is critical to improving learning outcomes and increasing the competence of future midwives. Without these foundational capacities, even well-designed curricula may not be delivered effectively [30, 32–34]. Thus, without addressing these gaps in educators’ preparation, efforts to improve midwifery education risk falling short of achieving the quality needed to ensure competent midwives.

Despite 46.9% (*n* = 53) of the respondents reporting combining clinical work with teaching, 39.8% (*n* = 45) expressed a need for training in clinical teaching using simulation. While many midwifery educators are formally meeting the expectations outlined in Category 6 *Maintaining midwifery practice competence* [8], they may struggle to translate their clinical competence into effective pedagogical practice. Results in other studies show that although midwifery educators are responsible for teaching and strengthening students’ clinical competence, they themselves receive limited training and support in pedagogy and curriculum design capabilities. This lack of pedagogical preparation makes it difficult for educators to translate their own clinical knowledge into effective teaching [6, 30, 33]. Maintaining clinical practice alone is therefore insufficient for effective teaching; educators also need targeted pedagogical support, including training in simulation-based methods, to adapt to modern standards of midwifery education [33, 35]. To sustain and further develop professional competence, aligned with the Category 6 *Maintaining midwifery practice competence* [8], there is a clear need for structured preparation in simulation pedagogy, including scenario design, debriefing and performance assessment.

This study reveals that the respondents mainly preferred professional development programmes, with most of them favouring “fully face-to-face” or “mostly face-to-face” blended formats. Preferences for intensive, short-duration courses reflect the constraints educators face in balancing teaching, clinical duties and professional development, pointing to the need for flexible and context-sensitive programme design. This is consistent with the results of Smith et al. [23] and a recent scoping review [35], which reported that midwives transitioning from clinical practice to academic roles often lack formal preparation for teaching. As a result, they required structured support and professional development to build their educational competencies. However, evidence suggests that traditional high-dose, low-frequency training models, commonly used in such short intensive courses, have only modest effects on provider performance, whereas repeated, practice-based learning and ongoing support demonstrate substantially greater improvements, in line with low-dose and high-frequency principles [36] This underscores the importance of ensuring that midwifery educators have access to adequate resources and flexible delivery modes, including face-to-face, online, and blended learning options, as emphasised in Category 5 *Resources* [8]. Supporting midwifery educators through such context-appropriate and evidence-informed approaches is essential for sustaining the quality of midwifery education programmes. In the priority area of research, a clear need for strengthened research competence was identified among respondents. This aligns directly with the ICM Global Standards Category 7 *Research*, which emphasises that educator development should build research skills and promote the integration of evidence into learning and teaching practices. Enhancing these competencies is therefore essential to ensuring that educational practices remain evidence-informed and aligned with international standards for midwifery education [8, 37]. More than one-third of the respondents reported a need to strengthen their skills in academic writing and research methodology. While many expressed a strong desire to contribute to evidence-based teaching, they simultaneously reported feeling unprepared to use and produce research. This finding is consistent with regional studies showing that midwifery educators often have limited research exposure and institutional support for research and scholarly activities [9, 14], underscoring the urgency of investment in this area. Strengthening research capability is foundational to evidence-informed teaching [38, 39] and contributes to the broader public health impact of midwifery [40, 41]. Investing in building research capacity among educators is essential for strengthening midwifery education and for advancing the profession.

While leadership competence in this study was identified as a key area for professional growth, previous research has shown that developing leadership competence should focus on enhancing communication, teamwork and problem-solving skills to create a positive working and learning environment [42, 43]. Developing midwifery educators’ knowledge in leadership is essential for educators to develop the skills, behaviours and attitudes needed to lead midwifery education [8, 14]. West et al. [3] argue that capacity building efforts must extend beyond the classroom to include strategic thinking, coordination and policy engagement. This is consistent with the Category 1 *Leadership*, which stipulates that midwifery educators should be supported to take on leadership roles in the design and implementation of midwifery education and quality improvement initiatives [8]. When educators are equipped to lead, not only in the classroom but also in strategic planning, coordination and policy processes, they are better positioned to drive sustainable context adopted improvements in education quality.

Although numerous training programmes for midwifery educators in Bangladesh, have been conducted since 2012 [16], these have largely remained short-term and disconnected from systematic career progression. The results of this study show that more than 50% of the respondents, regardless of profession, years of experience, or academic education, reported a need for professional development. This may indicate that the need for competence development is widely shared among midwifery educators and may point to the value of more structured and coordinated planning for professional development. As midwifery education expands and new institutions emerge, the educator profile is shifting, from nurses with postgraduate qualifications to midwives with bachelor’s degrees [17, 44], which may further reinforce the urgent need for more systematic strategies for professional development. To avoid “training without transfer,” educator development could be integrated into a broader career development framework that ensures newly acquired competencies are continuously applied, evaluated, and recognised. Integrating professional development with institutional quality assurance processes may also contribute to greater accountability for teaching and learning outcomes [8, 14]. Against this, the development of a more coordinated national strategy for the professional development of midwifery educators could be considered in alignment with national policy and the ICM Global Standards for Midwife Faculty Development [8] and linked to professional career pathway.

### Methodological considerations

While this study provides valuable insights into the professional development needs of midwifery educators in Bangladesh, some limitations should be acknowledged. The data were based on self-reported responses, which may be subject to recall bias or social desirability. Such potential bias could affect the internal validity [45] of the results, as reported needs may not perfectly correspond to actual competencies or institutional conditions. The cross-sectional design captures a single point in time and does not account for long-term changes in facilities’ needs or institutional capacity.

Although the sample size (*n* = 113) offers reasonable representation, the study may not fully capture regional or institutional variations within Bangladesh, particularly in rural or resource-constrained settings. Consequently, the external validity namely, the generalizability of the results to all midwifery education settings, may be limited. The study was also conducted in English, whereas most respondents are native Bengali speakers, which may have affected the clarity or depth of some responses. To support respondents understanding, the survey was reviewed with educators before distribution, and clarifications were available during data collection both in person and by telephone.

Another limitation is the decision not to analyse differences based on geographic distribution. As Bangladesh is still in the early stages of developing its midwifery education system and most educators have only a few years of experience teaching in midwifery programmes, such a subdivision was not expected to yield meaningful insights at this stage. However, this could be a relevant focus in future studies as the system and educators’ experience become more established. Future research should consider longitudinal designs and broader geographical sampling to further strengthen both internal and external validity, as well as the transferability of results across contexts [46].

## Conclusion

This study indicates three interlocking priorities for midwifery educators’ professional development in Bangladesh, pedagogical and curriculum design capabilities, research and leadership competence, while highlighting a need in clinical teaching using simulation. These needs appear across different educator roles and levels of experience, suggesting that gaps in educator preparation may be widespread. Embedding educator professional development within a national career framework may enhance the educator’s motivation and support continuous quality assurance in midwifery education in Bangladesh. The results point to the potential value of co-creating a national educators development programme, delivered through “fully face-to-face” or “mostly face-to-face” blended formats. Aligning these investments with quality improvement processes can accelerate educator capability, student competence and ultimately impact maternal and newborn health outcomes in Bangladesh.

## Supplementary Information

Supplementary Material 1: Annex 1. Midwifery educators’ needs survey questionnaire.

## Data Availability

Data used in the study will be provided on a reasonable request from the corresponding author.

## Abbreviations

UNFPA: United Nations Population Fund
Sida: Swedish International Development Cooperation Agency
DGNM: Directorate General of Nursing and Midwifery Services
