# Supplementary material for: Accelerating quality midwifery education: identifying educators’ professional development needs
Source: BMC Med Educ. 2026 Apr 29;26:695. doi: 10.1186/s12909-026-09322-4 (PMC13127029; doi:10.1186/s12909-026-09322-4)
Supplement: Supplementary file 1 — Supplementary Material 1: Annex 1. Midwifery educators’ needs survey questionnaire. [file 12909_2026_9322_MOESM1_ESM.docx]

| 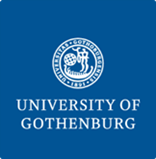 | SAHLGRENSKA ACADEMY  INSTITUTE OF HEALTH & CARE SCIENCES |
| --- | --- |

**Annex1: Faculty needs survey questionnaire**

I have read the Participant Information Sheet and consent to the data I enter being used as described in the information sheet

I have read the Participant Information Sheet and consent to the data I enter being used as described in the information sheet

1. What is your gender? M/F/other/prefer not to say
2. What is your age? (list)
3. What is your professional qualification? e.g. Certified Midwife; Nurse-Midwife; midwife; nurse but not midwife etc. (list)
4. What is your higher academic qualification (Diploma, bachelor’s degree, master’s degree, PhD)?
5. At which midwifery education institution do you work?
6. At which academic level of education do you teach? (Diploma, Bachelor, Master)
7. How long have you worked as a midwifery faculty member/teacher? (List)
8. Does your current role involve any of the following activities/responsibilities? Check all the boxes that apply

- Face-to-Face teaching (in-person, not online)
- Online or blended (mix of online and face-to-face) teaching
- Clinical teaching – Simulation in skills lab
- Clinical teaching – in clinical environment
- Curriculum design/Program and module development
- Program and Curriculum or Program implementation
- Research activities
- Writing manuscript for publication
- Institutional leadership
- Mentorship/Coaching
- Educational quality improvement activities

1. Please rank the activities/responsibilities in order of proportion of your time (with 1 being the most amount of your time), use the N/A if you are not involved in certain activities.

- Face-to-Face teaching
- Online or blended teaching
- Clinical teaching – Simulation
- Clinical teaching – In clinical environment
- Curriculum design/Program and module development
- Curriculum or Program and module implementation
- Research activities
- Writing manuscript for publication
- Institutional leadership
- Mentorship/Coaching
- Educational quality improvement activities

Capacity strengthening (Likert)

1. I need to strengthen my capacity in face-to-face teaching
2. I need to strengthen my capacity in blended/online teaching
3. I need to strengthen my capacity in clinical teaching using simulation
4. I need to strengthen my capacity in clinical teaching in the clinical environment
5. I need to strengthen my capacity in midwifery theory
6. I need to strengthen my capacity in midwifery clinical practice
7. I need to strengthen my capacity in curriculum design/program and module development
8. I need to strengthen my capacity in mentoring/coaching students
9. I need to strengthen my capacity in the conduct of research
10. I need to strengthen my capacity in writing manuscripts for publication
11. I need to strengthen my capacity in management and leadership
12. I need to strengthen my capacity in educational quality improvement activities
13. In addition to roles mentioned above, are there other areas of professional development you need to strengthen your role as a midwifery faculty? (free text)
14. Please list your top three areas/topics you need to strengthen, to assist you in your faculty role (1-3)
15. Have you had access to any formal Faculty Development programs (online, face-to face, blended training) in the past five (5) years (Yes/No)
16. If you have had access to formal Faculty Development in the last five (5) years - who provided the program/module?

Check all that apply. (List)

- My education institution (School/University) /government e.g. DGNM/BNMC provides formal development program/module
- Programs or modules provided by external/international universities and/or by UNFPA- please provide detail in comment section
- Not Applicable - no access to formal faculty development programs or module in last five (5) years
- Other (please specify)

1. Based on your needs: What would be your preferred delivery mode of a program or module of faculty development (1 is your most preferred option and 4 the least preferred)

- Online only
- Blended - mostly online with limited face-to-face
- Blended - mostly face-to-face with limited online
- Face-to-Face only

Please explain why the top option you chose for Q21 is your most preferred (Open text)

1. Based on your needs: what would be the preferred number of days for a program of faculty development?
2. What would be your preferred format/schedule for a XX- day (face-to-face or online) or equivalent program of faculty development (1 is your most preferred option and 4 your least preferred)

- Single point-in-time 10-day intensive program (over 2 weeks)
- One day each week for 10 weeks
- Half day every two weeks over a period of 6 months
- One day each month for 10 months

1. Please tell me why the top option you chose for Q23 is your most preferred
2. Do you currently work in midwifery clinical practice? (Yes/No)
3. When did you last spend time working as service provider in the clinical area as a midwife or nurse-midwife/certified midwife? (List)
4. If you are currently unable or not supported to work in the clinical area, please indicate if you would be interested in being contacted for follow-up research on the 'enablers and barriers to maintenance of clinical skills/practice for Faculty' - Participant Information Sheet (Yes/No)
5. Please provide any further comment that may help us determine your needs for developing and strengthening the work you do as midwifery faculty (Free Text)
